# Laboratory biomarkers and radiographic osteolysis after total knee arthroplasty: a retrospective pilot study

**DOI:** 10.3389/fsurg.2026.1835357

**Published:** 2026-05-22

**Authors:** Lifei Wang, Hefang Xiao, Jinming Liu, Haotian He, Bao Xian, Bin Geng, Yayi Xia

**Affiliations:** 1 Department of Orthopaedics, Lanzhou University Second Hospital, Lanzhou, Gansu, China; 2 Orthopedic Clinical Research Center of Gansu Province, Lanzhou University Second Hospital, Lanzhou, Gansu, China; 3 Intelligent Orthopedic Industry Technology Center of Gansu Province, Lanzhou University Second Hospital, Lanzhou, Gansu, China

**Keywords:** total knee arthroplasty, osteolysis, leukocyte count, risk factors, uric acid, creatinine, FIB-4 index

## Abstract

**Purpose:**

This study aimed to identify early risk factors for periprosthetic osteolysis after total knee arthroplasty (TKA) and establish clinically useful predictive biomarkers.

**Methods:**

A retrospective analysis was conducted on patients who underwent TKA at our institution between January 1, 2018, and October 31, 2023. Initially, 397 patients were screened, and 375 met the inclusion criteria after applying strict eligibility standards. Patients were categorized into an osteolysis group (*n* = 17) and a non-osteolysis group (*n* = 358). Data on baseline characteristics (age, gender, BMI, diabetes, and hypertension history) and postoperative laboratory results were collected. Logistic regression analyses identified independent risk factors for osteolysis, with subgroup analyses also performed.

**Results:**

Multivariable logistic regression analysis showed that each 1-unit increase in leukocyte count (OR = 1.36, 95% CI: 1.13–1.65, *P* = 0.001) and each 1-unit increase in FIB-4 index (OR = 1.94, 95% CI: 1.20–3.14, *P* = 0.007) were associated with higher odds of osteolysis, whereas each 1-unit increase in the uric acid-to-creatinine ratio (UACR) was associated with lower odds of osteolysis (OR = 0.57, 95% CI: 0.36–0.88, *P* = 0.012). Subgroup analyses suggested that the strength and significance of these associations varied by sex, age, diabetes status, and hypertension status.

**Conclusion:**

This study demonstrates that leukocyte count, FIB-4 index, and UACR are independent risk factors for early periprosthetic osteolysis after TKA. These findings may assist in the early identification and management of high-risk patients, thereby reducing postoperative complications and improving patient outcomes.

## 1 Introduction

Periprosthetic osteolysis remains one of the major causes of failure after total knee arthroplasty (TKA). Severe osteolysis can lead to prosthetic loosening and dislocation (1, 2).This not only directly results in suboptimal surgical outcomes or treatment failure, but also presents significant challenges for revision procedures (3, 4). With the increasing volume of TKA procedures, osteolysis has become one of the main complications affecting long-term prosthesis survival and postoperative function (5).In recent years, as TKA has been increasingly applied to patients with end-stage joint diseases such as knee osteoarthritis, more attention has been given to the management and prevention of postoperative complications.

Osteolysis is thought to be driven primarily by chronic local inflammatory responses to wear debris, particularly polyethylene particle (6–8). Wear particles released from the implant can be recognized by immune cells, especially macrophages, which then produce pro-inflammatory mediators, promote osteoclastogenesis, and ultimately lead to progressive periprosthetic bone resorptio (6, 7, 9). Recent reviews further suggest that this process involves not only the macrophage–osteoclast axis, but also a broader inflammatory cell infiltrate and complex osteoimmune interactions within the periprosthetic microenvironme (6–8). In addition to these biological mechanisms, osteolysis may also be influenced by broader host-related factors. Emerging evidence suggests that liver fibrosis-related metabolic burden and disturbances in renal or uric acid metabolism may be linked to bone remodeling and bone loss in other clinical settings, raising the possibility that hepatic and renal function could also be relevant to periprosthetic osteolysi (10–13).

Imaging modalities such as plain radiography, CT, and MRI are commonly used to evaluate osteolysis after TKA. Among them, conventional radiography remains the most widely used first-line examination because of its accessibility and low cost, but its sensitivity for early osteolytic lesions is limited (14, 15). Although CT and MRI can improve lesion detection and better define lesion size, location, cortical involvement, and surrounding soft tissue changes, they are not routinely performed in all patients during long-term follow-u (15). As a result, early radiographic osteolysis may still be difficult to identify in a timely manne.

While chronic inflammation and metabolic disturbances are increasingly recognized as relevant to osteolysis, simple and accessible laboratory markers for early risk assessment after TKA remain lacking. Previous studies have explored a variety of candidate biomarkers for aseptic loosening, but no widely accepted marker has yet entered routine clinical use (16). Therefore, in this retrospective pilot study, we screened patients for early-stage radiographic osteolysis after TKA and systematically evaluated its associations with routine laboratory biomarkers. The aim was to identify clinically accessible markers that may assist in early risk stratification and postoperative monitoring.

## 2 Materials and methods

### 2.1 Study design and patients

This was a single-center retrospective study approved by the institutional ethics committee. Patients who underwent total knee arthroplasty (TKA) between January 1, 2018, and October 31, 2023, were screened through the hospital medical record system. A total of 397 cases were initially identified.

Periprosthetic osteolysis was diagnosed on follow-up radiographs based on radiolucent lines >2 mm around the prosthesis, focal bone defects, or loss of trabecular bone structure compared with earlier postoperative radiographs. Early postoperative and approximately 3-month radiographs served as baseline reference images, and radiographs obtained at 1 year or later were used for outcome assessment. Only osteolysis without visible prosthetic loosening or displacement was classified as early-stage osteolysis. All radiographs were independently assessed by two orthopedic surgeons, and cases were included only when both reviewers agreed on the radiographic findings. In cases of disagreement, the final classification was determined by a senior orthopedic surgeon. Because of the retrospective design of the study, formal blinding to all clinical and laboratory information was not implemented. To ensure the completeness and consistency of the dataset, predefined inclusion and exclusion criteria were applied. Eligible patients were required to have standard anteroposterior and lateral radiographs of the operated knee obtained in the early postoperative period and at approximately 3 months after surgery, which served as baseline and early follow-up imaging. In addition, at least one radiographic follow-up assessment at 1 year or later, together with corresponding laboratory test results obtained during the follow-up period, was required for evaluation of radiographic osteolysis and its association with laboratory biomarkers. Only early-stage osteolysis cases, defined as radiographic osteolysis without visible prosthetic loosening or displacement, were included. Patients were excluded if follow-up laboratory data or imaging were unavailable n=22, or if revision surgery had been performed. After screening, 375 patients met the eligibility criteria, including 17 patients with radiographically confirmed osteolysis and 358 without osteolysis.

Baseline demographic and clinical variables were extracted from the records, including age, sex, body mass index (BMI), follow-up interval, history of diabetes (coded as 0 = no diabetes, 1 = diabetes without complications, 2 = diabetes with complications), and history of hypertension 0=no,1=yes. Full baseline characteristics are presented in Table 1. In addition, routine hematological and serological parameters from postoperative follow-up were collected for statistical analysis.

**Table 1:** Baseline characteristics of the study population (*n* = 375).

Variable	Total (*n* = 375)	Non-osteolysis (*n* = 358)	Osteolysis (*n* = 17)	*P*-value
Age, mean ± SD (years)	63.00 ± 9.49	62.74 ± 9.39	68.47 ± 10.18	0.015
Sex, *n* (%)				1.000
Men	85 (22.67%)	81 (22.63%)	4 (23.53%)	
Women	290 (77.33%)	277 (77.37%)	13 (76.47%)	
BMI, mean ± SD (kg/m^2^)	25.36 ± 3.61	25.31 ± 3.62	26.48 ± 3.28	0.193
Diabetes status, *n* (%)				0.008
No diabetes	330 (88.00%)	318 (88.83%)	12 (70.59%)	
With diabetes, no complications	35 (9.33%)	33 (9.22%)	2 (11.76%)	
With diabetes + complications	10 (2.67%)	7 (1.96%)	3 (17.65%)	
Hypertension, *n* (%)				0.778
No	186 (49.60%)	177 (49.44%)	9 (52.94%)	
Yes	189 (50.40%)	181 (50.56%)	8 (47.06%)	

Data are presented as mean ± standard deviation or number (%). Statistical significance was evaluated using *t*-test for continuous variables and chi-square test for categorical variables.

### 2.2 Statistical analysis

Continuous variables were expressed as mean ± standard deviation (x¯ ± s), while categorical variables were presented as frequencies and percentages (*n*, %). Comparisons between groups were performed using the Student's *t*-test for continuous variables and chi-square test for categorical variables to evaluate baseline comparability.

To explore associations with periprosthetic osteolysis, univariate logistic regression analysis was first conducted to screen potential predictors. Variables with *P*-values < 0.05 in univariate analysis were then entered into a multivariate logistic regression model to identify independent risk factors. A bidirectional stepwise regression approach was used to optimize model performance. This method combines forward selection and backward elimination, aiming to balance model complexity and predictive accuracy. The stepwise procedure was as follows: Initialization: The model was initialized either from an empty model (adding variables sequentially) or from a full model (removing variables one by one). Forward selection: Variables with *P*-values < 0.05 and the greatest incremental improvement to the model were added. Backward elimination: Variables with *P*-values ≥ 0.05 and minimal contribution were removed. Iteration continued until all variables in the final model had *P*-values < 0.05. All statistical analyses were conducted using the Statistical analyses were conducted using the Storm Statistics Platform (Zstats, Beijing, China; https://www.zstats.net), R version 4.3.3 (R Foundation for Statistical Computing, Vienna, Austria), and IBM SPSS Statistics version 27.0 (IBM Corp., Armonk, NY, USA). Because several inflammation-related variables, such as leukocyte count, monocyte count, neutrophil count, SIRI, and PIV, are likely to capture overlapping information on systemic inflammation, we first assessed collinearity among them using Spearman correlation analysis and variance inflation factors (VIFs). Given the small number of osteolysis events, these highly correlated markers were not included in the same multivariable model. We therefore constructed several alternative parsimonious models, each including one inflammation-related marker together with FIB-4 index and UACR. The final model was chosen by considering collinearity, model fit, discrimination, and clinical interpretability.

## 3 Results

A total of 375 patients were included in this study. Among them, 358 patients (95.47%) did not develop radiographic evidence of periprosthetic osteolysis, while 17 patients (4.53%) were diagnosed with early-stage osteolysis. The average age of patients without osteolysis was 62.74 years, while that of those with osteolysis was 68.47 years; the difference was statistically significant (*t* = −2.45, *P* = 0.015).

In terms of BMI, the non-osteolysis group had a mean of 25.31 kg/m^2^, and the osteolysis group had a mean of 26.48 kg/m^2^; however, the difference was not statistically significant (*t* = −1.30, *P* = 0.193).

Regarding diabetes status, in the non-osteolysis group, 88.83% had no history of diabetes, 9.22% had diabetes without complications, and 1.96% had diabetes with complications. In the osteolysis group, 70.59% had no diabetes, 11.76% had uncomplicated diabetes, and 17.65% had diabetes with complications. The distribution difference was statistically significant (*P* = 0.008).There was no significant difference in hypertension distribution between the two groups (*χ*^2^ = 0.08, *P* = 0.778), nor in sex distribution (*χ*^2^ = 0.00, *P* = 1.000). Detailed results are shown in Table 1.

### 3.1 Univariate logistic regression

In the univariate analysis, several laboratory and clinical parameters were significantly associated with an increased risk of periprosthetic osteolysis. These included leukocyte count, monocyte count, neutrophil count, SIRI (Systemic Inflammation Response Index), PIV (Pan-Immune-Inflammation Value), age, diabetes status, FIB-4 index, serum creatinine, and direct bilirubin (all *P* < 0.05). Notably, UACR (uric acid to creatinine ratio) and ALT/AST ratio emerged as protective factors, both demonstrating inverse associations with osteolysis risk. In contrast, BMI, SII, NLR, and several other inflammatory markers showed no statistically significant correlation. Detailed regression results are presented in Table 2.

**Table 2:** Univariate logistic regression variables.

Significant Variables (*P* < 0.05)		
Variable	OR (95% CI)	*P* value
Leukocyte count (WBC)	1.68 (1.20–2.36)	0.003*
Monocyte count	1.62 (1.18–2.23)	**0** **.** 003*
SIRI (Systemic Inflammation Response Index)	1.45 (1.13–1.86)	**0** **.** 003*
Diabetes group/status	2.93 (1.43–6.02)	**0** **.** 003*
Neutrophil count	1.60 (1.17–2.19)	**0** **.** 004*
PIV (pan-immune-inflammation value)	1.40 (1.09–1.81)	**0** **.** 010*
Age	2.25 (1.21–4.16)	**0** **.** 010*
FIB-4 index	1.55 (1.10–2.16)	**0** **.** 011*
UACR (Uric Acid to Creatinine Ratio)	0.47 (0.26–0.87)	**0** **.** 016*
Creatinine	1.39 (1.06–1.83)	**0** **.** 016*
ALT/AST ratio	0.48 (0.25–0.94)	**0** **.** 033*
Direct bilirubin	1.53 (1.02–2.28)	**0** **.** 038*
Non-significant Variables (*P* ≥ 0.05)		
Variable	OR (95% CI)	*P* value
Cystatin C	1.33 (0.99–1.77)	0.055
Neutrophil percentage	1.53 (0.96–2.43)	0.071
SII (Systemic Immune-Inflammation Index)	1.31 (0.97–1.76)	0.074
Adenosine deaminase	1.35 (0.96–1.90)	0.082
NLR (neutrophil/lymphocyte ratio)	1.27 (0.97–1.68)	0.086
Lymphocyte percentage	0.66 (0.41–1.07)	0.091
Mean platelet volume (MPV)	1.44 (0.93–2.22)	0.104
APRI (Aspartate aminotransferase-to-platelet ratio index)	1.29 (0.94–1.77)	0.110
Urea/creatinine ratio	0.61 (0.34–1.12)	0.111
Large platelet ratio	1.43 (0.92–2.23)	0.113
Indirect bilirubin	0.61 (0.33–1.14)	0.120
Chloride	0.70 (0.44–1.10)	0.123
Alkaline phosphatase (ALP)	0.54 (0.23–1.26)	0.152
BMI (body mass index)	1.41 (0.85–2.35)	0.187
LDL cholesterol	0.73 (0.43–1.21)	0.219
Creatine kinase-MB	0.64 (0.31–1.32)	0.225
BMI grade	1.39 (0.79–2.44)	0.252
Sodium	1.34 (0.81–2.23)	0.254
Eosinophil percentage (EDTA anticoagulated)	0.67 (0.34–1.34)	0.258
Alpha-L-fucosidase	0.73 (0.42–1.26)	0.260
Weight	1.30 (0.81–2.10)	0.276
HALP score	1.25 (0.83–1.89)	0.282
Monoamine oxidase	0.75 (0.44–1.27)	0.284
ALBI grade	1.48 (0.72–3.04)	0.284
Triglycerides	0.70 (0.36–1.37)	0.302
Magnesium	1.27 (0.80–2.02)	0.307
Red blood cell count	0.79 (0.50–1.25)	0.313
Platelet distribution width	1.25 (0.80–1.97)	0.325
Immature granulocyte count	1.15 (0.86–1.54)	0.341
C-reactive protein (CRP)	0.43 (0.08–2.46)	0.344
CAR (C-reactive protein/albumin ratio)	0.45 (0.08–2.48)	0.362
Lipoprotein(a)	0.65 (0.25–1.71)	0.380
LMR (lymphocyte/monocyte ratio)	0.76 (0.41–1.40)	0.380
Amylase	1.20 (0.79–1.81)	0.395
Hemoglobin	0.82 (0.52–1.30)	0.404
Hematocrit	0.83 (0.52–1.30)	0.406
Blood urea	1.18 (0.78–1.77)	0.437
GGT/ALP ratio	1.18 (0.78–1.79)	0.439
Complement C1q	0.81 (0.48–1.38)	0.440
Aspartate aminotransferase (AST)	1.14 (0.80–1.63)	0.472
Uric acid/fasting glucose ratio	0.82 (0.48–1.40)	0.476
ALBI score	1.21 (0.71–2.05)	0.488
Follow-up interval (days)	1.14 (0.75–1.75)	0.538
Prealbumin	0.83 (0.47–1.49)	0.540
Potassium	1.16 (0.71–1.90)	0.544
GFR (glomerular filtration rate)	0.85 (0.51–1.44)	0.552
Red cell distribution width-SD	1.13 (0.75–1.72)	0.560
Mean corpuscular volume (MCV)	1.15 (0.71–1.87)	0.577
Albumin/globulin ratio	0.87 (0.53–1.43)	0.578
Bile acid	1.12 (0.74–1.68)	0.598
Apolipoprotein A-I	1.13 (0.70–1.83)	0.606
Basophil percentage (EDTA anticoagulated)	6.19 × 10^−12^	
(2.09 × 10^−55^–1.83 × 10^+32^)	0.613	
Red cell distribution width-CV	0.87 (0.51–1.49)	0.620
Lactate dehydrogenase (LDH)	1.12 (0.71–1.78)	0.626
Total cholesterol	0.88 (0.54–1.46)	0.629
Alpha-hydroxybutyrate dehydrogenase	1.12 (0.70–1.78)	0.632
Mean corpuscular hemoglobin (MCH)	1.13 (0.68–1.87)	0.634
Gamma-glutamyltransferase (GGT)	0.79 (0.28–2.18)	0.644
PALBI score	1.12 (0.68–1.86)	0.649
Creatine kinase	1.07 (0.71–1.61)	0.734
Apolipoprotein B	0.92 (0.56–1.51)	0.735
Lymphocyte count	1.07 (0.67–1.72)	0.772
Eosinophil count (EDTA anticoagulated)	0.92 (0.53–1.60)	0.777
Hypertension	0.87 (0.33–2.30)	0.778
PAR (platelet/albumin ratio)	0.93 (0.57–1.53)	0.782
Phosphate	1.06 (0.66–1.71)	0.811
Globulin	1.06 (0.66–1.70)	0.821
GPS	0.84 (0.19–3.79)	0.821
Basophil count (EDTA anticoagulated)	1.05 (0.66–1.68)	0.831
Uric acid/albumin ratio	0.95 (0.57–1.57)	0.835
BMI group	1.08 (0.51–2.30)	0.837
Height	0.87 (0.22–3.46)	0.839
Platelet count	0.95 (0.58–1.55)	0.843
Calcium	0.96 (0.61–1.51)	0.858
Monocyte percentage	1.04 (0.64–1.67)	0.886
Mean corpuscular hemoglobin concentration (MCHC)	0.97 (0.59–1.57)	0.887
PLR (platelet/lymphocyte ratio)	0.97 (0.59–1.59)	0.898
Fasting blood glucose	1.03 (0.65–1.64)	0.904
HDL cholesterol	1.03 (0.64–1.66)	0.905
Total protein	1.03 (0.63–1.67)	0.918
Alanine aminotransferase (ALT)	0.98 (0.59–1.62)	0.922
Uric acid	0.98 (0.60–1.60)	0.924
PNI (prognostic nutritional index)	1.02 (0.63–1.67)	0.926
Homocysteine	0.98 (0.59–1.61)	0.926
Serum iron	0.98 (0.60–1.60)	0.931
Sex	0.95 (0.30–2.99)	0.931
Albumin	0.98 (0.61–1.60)	0.943
Immature granulocyte percentage	0.98 (0.59–1.63)	0.945
Plateletcrit	1.01 (0.62–1.65)	0.958
Total bilirubin	0.99 (0.61–1.61)	0.960
GPR (Gamma-glutamyl transpeptidase to platelet ratio)	0.99 (0.60–1.64)	0.979
Uric acid-CRP index	1.00 (0.61–1.63)	0.988
Uric acid/HDL ratio	1.00 (0.62–1.63)	0.997

OR, odds ratio; CI, confidence interval.

Bold values indicate statistically significant results.

*P < 0.05.

Several inflammation-related variables were significant in the univariable analysis, including leukocyte count, monocyte count, neutrophil count, SIRI, and PIV. Because these markers are biologically related and likely capture overlapping inflammatory information, collinearity was further assessed. Strong inter-correlations were observed, particularly between leukocyte count and neutrophil count, and between SIRI and PIV (Supplementary Tables S5–S6).

### 3.2 Multivariate logistic regression

Multivariable logistic regression with bidirectional stepwise selection identified leukocyte count, UACR, and FIB-4 index in the final model. After adjustment, higher leukocyte count and higher FIB-4 index were associated with higher odds of osteol ysis, whereas higher UACR was associated with lower odds. Specifically, each 1-unit increase in leukocyte count was associated with a 36% increase in the odds of osteolysis, each 1-unit increase in UACR was associated with a 43% decrease in the odds of osteolysis, and each 1-unit increase in FIB-4 index was associated with an approximately 94% increase in the odds of osteolysis. Full model outputs are shown in Table 3.

**Table 3:** Multivariable logistic regression analysis results: factors associated with the occurrence of periprosthetic osteolysis.

Variable	β	OR	95%CI	*P* value
FIB-4 Index	0.661230	1.937	1.196–3.136	0.0072
UACR (Uric Acid to Creatinine Ratio)	−0.570317	0.565	0.362–0.884	0.0124
Leukocyte count (WBC)	0.309171	1.362	1.127–1.646	0.0014

OR, odds ratio; CI, confidence interval.

Several inflammation-related variables were significant in the univariable analysis and appeared to capture overlapping information. Collinearity analysis showed substantial inter-correlation among these markers. In the subsequent comparison of alternative parsimonious models, the WBC-based model showed the most favorable overall performance, with the lowest AIC, the highest AUC, and the highest specificity while maintaining acceptable sensitivity. Although the neutrophil-based model showed higher sensitivity, this gain was accompanied by lower specificity and less favorable overall model fit, suggesting that it may be more suitable for screening-oriented assessment rather than as the primary model for overall risk stratification. These model outputs are shown in Table 4. These findings may support the use of leukocyte count as the representative inflammation-related marker in the primary multivariable model.

**Table 4:** Univariate logistic regression variables.

Variables	*P*	AUC	AUC 95% CI	Sensitivity	Specificity
WBC + FIB4 + UACR	0.0014	0.811	0.697–0.904	0.647	0.880
Monocyte + FIB4 + UACR	0.0019	0.770	0.646–0.875	0.647	0.844
Neutrophil + FIB4 + UACR	0.0041	0.802	0.686–0.894	0.882	0.687
PIV + FIB4 + UACR	0.0023	0.799	0.682–0.899	0.765	0.737

AUC, area under the receiver operating characteristic curve; CI, confidence interval; WBC, white blood cell count; FIB-4, fibrosis-4 index; UACR, uric acid-to-creatinine ratio; PIV, pan-immune-inflammation value.

Overall, these findings suggest that systemic inflammatory status, metabolic factors, and liver fibrosis-related indices may be associated with early radiographic osteolysis, although these associations should be interpreted cautiously.

#### 3.2.1 ROC curve analysis

**Figure 1:**
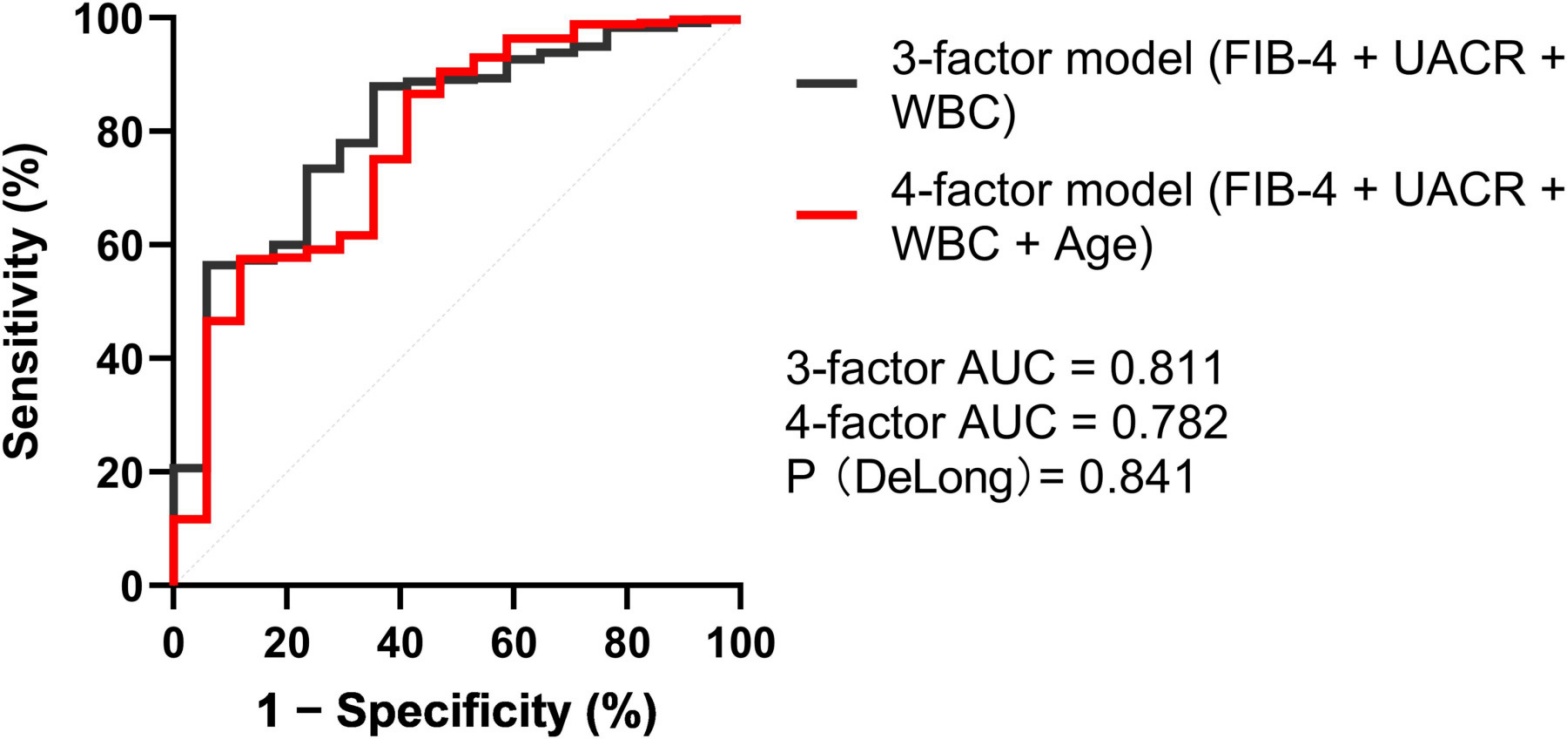
Receiver operating characteristic (ROC) curves of the 3- and 4-factor models for predicting periprosthetic osteolysis after total knee arthroplasty (TKA). The 3-factor model included FIB-4, uric acid-to-creatinine ratio (UACR), and leukocyte count (WBC), whereas the 4-factor model additionally included age. The 3-factor model achieved an AUC of 0.811 and the 4-factor model an AUC of 0.782; the difference between AUCs was not significant (DeLong test, *P* = 0.841). The dashed diagonal line indicates no-discrimination performance (AUC = 0.5).

ROC curve analysis of the primary multivariable logistic regression model showed acceptable discrimination, with an AUC of 0.811 (95% CI: 0.702–0.918, *P* < 0.001). At the optimal cutoff probability of 0.070, the 3-factor model achieved a sensitivity of 64.7% and a specificity of 88.0%. For comparison, the ROC curves of the 3-factor model (FIB-4, UACR, and WBC) and the age-adjusted 4-factor model (FIB-4, UACR, WBC, and age) were displayed in the same figure. The AUCs were similar (0.811 vs. 0.782), and DeLong's test did not indicate a significant difference (*P* = 0.841), suggesting limited incremental discrimination from adding age (Figure 1).

#### 3.2.2 Internal validation

Bootstrap internal validation was performed with 1,000 resamples. The apparent AUC was 0.8107. The estimated optimism was 0.0317, yielding an optimism-corrected AUC of 0.7790, which indicates acceptable discrimination after correction. The apparent Brier score was 0.0402, and the optimism-corrected Brier score was 0.0433. Calibration intercept and slope were 0.0000 and 1.0000 in the apparent model; after correction, the calibration intercept was −0.3246 and the calibration slope was 0.8667. The corrected calibration slope suggests mild overfitting, with slightly optimistic risk estimates. External validation in an independent cohort is needed to confirm generalizability.

### 3.3 Subgroup analyses

Subgroup analyses were performed according to sex, age, diabetes status, and hypertension status; however, these results should be interpreted cautiously because of the small number of osteolysis events within individual strata.

#### 3.3.1 Sex-based subgroup analysis

In women, higher UACR was associated with lower odds of osteolysis OR=0.31,P=0.046, whereas leukocyte count showed a borderline association P=0.053 and FIB-4 index was not statistically significant. In men, higher leukocyte count OR=1.39,P=0.006 and higher FIB-4 index OR=2.63,P=0.001 were associated with higher odds of osteolysis, whereas UACR did not reach statistical significance P=0.101. These findings may suggest sex-related differences in the relative contribution of inflammatory and metabolic markers, but they should be regarded as exploratory.

#### 3.3.2 Age-based subgroup analysis

Among patients aged ≥65 years, leukocyte count OR=1.58,P=0.003, FIB-4 index OR=2.11,P=0.049, and UACR OR=0.43,P=0.011 were all associated with osteolysis risk. In contrast, none of these predictors reached statistical significance in the 50–65 years or ≤50 years subgroups. Given the limited number of events in the younger strata, these negative findings may reflect insufficient statistical power rather than the absence of an association.

#### 3.3.3 Diabetes-based subgroup analysis

In the non-diabetic subgroup (*n* = 330; osteolysis events = 12), leukocyte count OR=1.34,P=0.006 and FIB-4 index OR=1.72,P=0.048 were associated with osteolysis, whereas UACR was not statistically significant P=0.135. In the diabetic subgroup (*n* = 45; osteolysis events = 5), FIB-4 index remained associated with osteolysis risk OR=5.06,P=0.035, while UACR showed a borderline association OR=0.23,P=0.058 and leukocyte count was not statistically significant P=0.180. Because of the very small number of events in the diabetic subgroup, these estimates should be interpreted with particular caution.

#### 3.3.4 Hypertension-based subgroup analysis

In hypertensive patients, leukocyte count OR=1.42,P=0.008 and UACR OR=0.38,P=0.009 were associated with osteolysis, whereas FIB-4 index was not statistically significant. In non-hypertensive patients, leukocyte count OR=1.40,P=0.037 and FIB-4 index OR=2.18,P=0.027 were associated with osteolysis, whereas UACR was not statistically significant. These subgroup-specific patterns may indicate potential heterogeneity across clinical strata, although confirmation in larger cohorts is needed.

Overall, the subgroup analyses suggested that the associations of leukocyte count, UACR, and FIB-4 index with osteolysis were not entirely uniform across patient subgroups. However, given the limited number of osteolysis events, these analyses should be considered exploratory and hypothesis-generating. Subgroup results are summarized in Supplementary Tables S1–S4.

## 4 Discussion

This study systematically analyzed the clinical and laboratory parameters of patients after total knee arthroplasty (TKA) and identified leukocyte count, FIB-4 index, and uric acid to creatinine ratio (UACR) as independent predictors of early periprosthetic osteolysis. These variables remained significant even after adjustment for confounders in the multivariate logistic model. Subgroup analyses further indicated that some risk factors may be modulated by sex, age, and comorbidities such as diabetes and hypertension. These findings suggest that osteolysis is a multifactorial complication involving inflammatory, metabolic, and systemic pathways, underscoring the need for individualized monitoring and intervention strategies.

In our cohort, higher leukocyte count was associated with radiographic osteolysis, which may suggest a possible contribution of systemic inflammation to the development of periprosthetic osteolysis. In the setting of TKA, this association may be biologically plausible because periprosthetic osteolysis is driven by chronic particle-induced sterile inflammation, in which macrophage activation, inflammatory mediator release, and osteoclastogenesis play central roles. Bone metabolism and the hematopoietic system are closely interlinked, and numerous studies have shown that circulating leukocyte levels reflect changes in bone remodeling (17–20). For instance, Valderrábano et al. found that elderly men with rapid hip bone loss (>0.5% per year) were more likely to exhibit anemia, elevated neutrophil counts, and reduced lymphocyte levels (17). Kristjansdottir et al. reported in ambulatory elderly men from the MrOS Sweden cohort that neutrophil count was negatively associated with total body and total hip bone mineral density, and that the association with total hip BMD remained significant after multivariable adjustment (18). Recent single-cell RNA sequencing has shown that osteoporotic patients exhibit shifts in monocyte subsets, with upregulation of inflammation and osteoclast activation pathways, especially CD16⁺ terminal monocytes (20). In addition, studies in female populations have shown that both red and white blood cell counts are positively correlated with bone mineral density and bone microarchitecture parameters, suggesting that optimal bone health depends on a well-functioning hematopoietic and immune microenvironment (18). As an additional example from an orthopedic setting, Moldovan described sterile inflammation as a non-infectious inflammatory response triggered by surgical tissue injury and showed that postoperative hematologic indices, including NLR, PLR, MLR, SII, SIRI, and AISI, were associated with the magnitude of surgery-related inflammatory burden after fracture fixation, supporting the broader concept that routine blood-derived markers may reflect non-infectious inflammatory status in bone-related conditions (21). Recent meta-analytic evidence further supports a broader link between blood-derived immune-inflammatory indices and bone loss, with elevated NLR, PLR, MLR, and SII reported to be associated with increased osteoporosis risk (22). However, these findings should be interpreted cautiously, because most of the cited studies focused on osteoporosis, bone mineral density, or general bone loss in community-based populations rather than periprosthetic osteolysis after TKA. Therefore, they should be considered indirect rather than direct support for our findings.

Uric acid is the final product of purine nucleotide metabolism in the human body (23). Its extracellular antioxidant properties are believed to play a role in skeletal metabolism (24). Regarding the relationship between creatinine and bone mineral density (BMD), creatinine also exhibits antioxidant capacity *in vivo*, and several studies suggest that creatinine may serve as a protective factor for bone density. Serum uric acid levels have been shown to correlate positively with BMD, indicating that hyperuricemia may help prevent osteoporosis (25). In elderly individuals with normal renal function, serum creatinine has been reported to be positively associated with bone mineral density, while recent studies and dose-response meta-analytic evidence suggest that serum uric acid may also be positively related to bone mineral density and inversely related to osteoporosis risk (12, 13). In our study, the uric acid-to-creatinine ratio (UACR) was significantly associated with a reduced risk of periprosthetic osteolysis. Specifically, an increase of 1 unit in UACR was associated with a 43% decrease in osteolysis risk (OR = 0.565), suggesting that UACR may serve as a protective biomarker. Consistent with our findings, a large-scale NHANES-based study by Yu et al. (2025) also demonstrated a significant inverse association between UA/Cr and osteoporosis risk in older adults (OR = 0.83, 95% CI: 0.76–0.91, *P* < 0.001). Their quartile-based analysis further confirmed that individuals in higher UACR groups had a lower risk of osteoporosis, and the predictive performance of UA/Cr was superior to that of serum uric acid alone (26). However, the biological relevance of UACR to periprosthetic osteolysis remains uncertain, because prior studies mainly examined osteoporosis or bone metabolic status in broader populations rather than TKA-specific osteolysis.

An elevated FIB-4 index, a non-invasive marker originally developed to estimate liver fibrosis, was also associated with higher odds of periprosthetic osteolysis in our study. The FIB-4 index has been widely used to assess the degree of liver fibrosis in patients with chronic liver disease. In recent years, the relationship between liver fibrosis (including non-invasive indicators such as FIB-4) and reduced bone mineral density or increased osteoporosis risk has been the subject of ongoing debate. Several case-control and cross-sectional studies (e.g., by Barchetta and Kim) have found that higher degrees of liver fibrosis are significantly associated with lower bone density and increased osteoporosis risk (27, 28). However, other studies, such as multicenter analyses based on the NHANES database, reported that after adjusting for confounding metabolic factors like BMI and diabetes, the association between liver fibrosis and BMD or osteoporosis was no longer significant (29).These discrepancies may be explained by differences in study populations, disease status (e.g., obesity, diabetes), comorbidities, and assessment methods. A large population-based study from the Bushehr Elderly Health Program found that FIB-4 scores were significantly higher in individuals with osteoporosis, and even after adjustment for confounders, FIB-4 remained negatively correlated with hip and femoral neck BMD as well as trabecular bone score. Moreover, FIB-4 was significantly associated with an increased risk of osteoporosis (OR = 2.12 in women; OR = 1.37 in men) (27). It has been proposed that the link between liver fibrosis and bone metabolism disorders may involve multiple pathological mechanisms, including systemic inflammation, insulin resistance, disruption of the RANKL/OPG system, and interactions involving gut microbiota and the “liver–bone axis” (11, 30–32). Against this background, our finding that FIB-4 was associated with radiographic osteolysis may suggest that liver fibrosis-related metabolic and inflammatory burden could be relevant to periprosthetic bone loss. However, because most previous studies focused on osteoporosis or bone mineral density rather than TKA-related osteolysis, this evidence should be considered indirect. Further studies are needed to clarify the role of FIB-4 in periprosthetic osteolysis.

Subgroup analyses suggested that the associations of leukocyte count, UACR, and FIB-4 index with osteolysis were not entirely uniform across sex, age, diabetes status, and hypertension status. In men, leukocyte count and FIB-4 index remained associated with osteolysis, whereas in women UACR showed the strongest signal. In patients aged ≥65 years, all three predictors were associated with osteolysis, while no significant associations were observed in younger strata. Similarly, FIB-4 appeared to remain associated with osteolysis in the diabetic subgroup, whereas leukocyte count and FIB-4 were more evident in non-diabetic patients. Different patterns were also observed according to hypertension status. These findings may suggest potential heterogeneity in the relative contribution of inflammatory, metabolic, and fibrosis-related markers across clinical subgroups. However, given the very small number of osteolysis events overall and within each subgroup, these results should be interpreted with particular caution and should be considered exploratory and hypothesis-generating rather than confirmatory. Larger studies are needed to determine whether these subgroup-specific patterns are reproducible.

This study has several limitations. Its single-center retrospective design may have introduced selection bias and residual confounding. The small number of osteolysis events limits the robustness of the multivariable and subgroup analyses. In addition, osteolysis was defined radiographically rather than by CT or MRI, so some small or occult lesions may have been missed. Laboratory testing was based on routine follow-up rather than a fully standardized schedule, and no external validation cohort was available. Therefore, these findings should be interpreted cautiously and require confirmation in larger prospective studies. In addition, radiographic assessment was not performed under formal blinded conditions, which may have introduced assessment bias.

## 5 Conclusion

In this retrospective pilot study, higher leukocyte count and FIB-4 index, as well as lower UACR, were associated with early radiographic periprosthetic osteolysis after total knee arthroplasty. These findings suggest that inflammatory status, metabolic factors, and liver fibrosis-related indices may be relevant to osteolysis risk and may have potential value for early risk stratification. However, given the single-center retrospective design, the small number of osteolysis events, and the exploratory nature of the subgroup analyses, these results should be interpreted cautiously. Further validation in larger, independent, and preferably prospective cohorts is needed before these biomarkers can be applied in routine clinical practice.

## Data Availability

The raw data supporting the conclusions of this article will be made available by the authors, without undue reservation.

## Supplementary material

Supplementary Table S1 Sex-based subgroup analysis.

Supplementary Table S2 Age-based subgroup analysis.

Supplementary Table S3 Diabetes-based subgroup analysis.

Supplementary Table S4 Diabetes status-based subgroup analysis.

Supplementary Table S5 Spearman correlation analysis of inflammation-related markers.

Supplementary Table S6 Variance inflation factor analysis of inflammation-related markers.
