# Supplementary material for: Laboratory biomarkers and radiographic osteolysis after total knee arthroplasty: a retrospective pilot study
Source: Front Surg. 2026 May 22;13:1835357. doi: 10.3389/fsurg.2026.1835357 (PMC13236959; doi:10.3389/fsurg.2026.1835357)
Supplement: Supplementary Table S1 — Sex-based subgroup analysis. [file Table1.docx]

**Supplementary Table 1. Gender Subgroup Analysis**

| **Variable** | **Men** | | **Women** | |
| --- | --- | --- | --- | --- |
|  | **OR(95%CI)** | **P** | **OR(95%CI)** | **P** |
| UACR | 0.54(0.29-1.01) | 0.05 | 0.23(0.05-1.00) | 0.05 |
| WBC | 1.58(1.20-2.08) | <0.01 | 1.66(0.98-2.83) | 0.06 |
| FIB-4 index | 3.10(1.65-5.82) | <0.01 | 1.08(0.38-3.05) | 0.88 |
| BMI | 1.28(1.03-1.60) | 0.03 | 1.36(0.80-2.30) | 0.26 |

OR: Odds Ratio, CI: Confidence Interval

**Supplementary Table 2. Age Subgroup Analysis**

| **Variable** | **≤50** | | **50-65** | | **≥65** | |
| --- | --- | --- | --- | --- | --- | --- |
|  | OR(95%CI) | P | OR(95%CI) | P | OR(95%CI) | P |
| UACR | 1.55(0.10-22.86) | 0.75 | 0.56(0.22-1.42) | 0.22 | 0.38(0.18-0.78) | <0.01 |
| WBC | 0.86(0.19-3.81) | 0.84 | 1.18(0.79-1.74) | 0.42 | 1.74(1.25-2.43) | <0.01 |
| FIB-4 index | 0.77(0.00-1648.99) | 0.95 | 1.20(0.32-4.47) | 0.79 | 2.33(0.96-5.61) | 0.06 |
| BMI | 1.24(0.71-2.17) | 0.45 | 1.10(0.83-1.46) | 0.50 | 1.32(1.00-1.74) | 0.05 |

OR: Odds Ratio, CI: Confidence Interval

**Supplementary Table 3. Diabetes Subgroup Analysis**

| **Variable** | **NoDiabetes** | | **Diabetes** | |
| --- | --- | --- | --- | --- |
|  | OR(95%CI) | P | OR(95%CI) | P |
| UACR | 0.64(0.37-1.11) | 0.11 | 0.13(0.02-0.96) | 0.05 |
| WBC | 1.43(1.14-1.78) | <0.01 | 1.91(0.81-4.53) | 0.14 |
| FIB-4 index | 1.84(1.04-3.26) | 0.04 | 6.83(1.08-43.06) | 0.04 |
| BMI | 1.23(1.01-1.50) | 0.04 | 1.50(0.76-2.94) | 0.24 |

OR: Odds Ratio, CI: Confidence Interval

**Supplementary Table 4. Hypertension Subgroup Analysis**

| **Variable** | **NoHypertension** | | **Hypertension** | |
| --- | --- | --- | --- | --- |
|  | OR(95%CI) | P | OR(95%CI) | P |
| UACR | 0.74(0.40-1.36) | 0.33 | 0.32(0.14-0.71) | <0.01 |
| WBC | 1.47(1.06-2.03) | 0.02 | 1.64(1.18-2.28) | <0.01 |
| FIB-4 index | 2.60(1.25-5.43) | 0.01 | 1.96(0.90-4.26) | 0.09 |
| BMI | 1.25(0.99-1.59) | 0.06 | 1.35(0.98-1.85) | 0.07 |

OR: Odds Ratio, CI: Confidence Interval

Supplementary Table 5

| **Variable** | **WBC** | **Monocyte** | **Neutrophil** | **SIRI** | **PIV** |
| --- | --- | --- | --- | --- | --- |
| **WBC** | 1.000 | 0.587 | 0.889 | 0.616 | 0.674 |
| **Monocyte** | 0.587 | 1.000 | 0.483 | 0.650 | 0.656 |
| **Neutrophil** | 0.889 | 0.483 | 1.000 | 0.792 | 0.794 |
| **SIRI** | 0.616 | 0.650 | 0.792 | 1.000 | 0.898 |
| **PIV** | 0.674 | 0.656 | 0.794 | 0.898 | 1.000 |

WBC: white blood cell count; SIRI: systemic inflammation response index. PIV: pan-immune-inflammation value.

Supplementary Table 6

| **Variable** | **VIF** | **Interpretation** |
| --- | --- | --- |
| Neutrophil | 20.33 | Severe |
| WBC | 16.28 | Severe |
| SIRI | 7.76 | High |
| PIV | 7.22 | High |
| Monocyte | 2.86 | Acceptable |

WBC: white blood cell count; SIRI: systemic inflammation response index. PIV: pan-immune-inflammation value.
